# Comparative analysis of the nutritional, biochemical and hematological parameters of pregnant women attending the University of Abuja Teaching Hospital, Nigeria

**DOI:** 10.37796/2211-8039.1237

**Published:** 2022-03-01

**Authors:** Anthony Uchenna Emeribe, Amos Dangana, Hezekiah Alkali Isa, Solomon Oloche Onoja, Theresa Ohunene Otu, Yakubu Ibrahim, Amilia Afzan Mohd Jamil, Justin Onyebuchi Nwofe, Moses D. Lugos, Dorcas Aliyu, Shamsuddeen Haruna, Mala Alhaji Baba Mallam, Saidu Maijiddah Aminu, Hadiza Yahaya, Silifat Oyewusi, Mustapha Bakare, Idris Nasir Abdullahi

**Affiliations:** a Department of Medical Laboratory Science, Faculty of Allied Medical Sciences, University of Calabar, Calabar, Nigeria; b Department of Medical Laboratory Services, University of Abuja Teaching Hospital, Gwagwalada, Abuja, Nigeria; c Department of Hematology, University of Abuja Teaching Hospital, Gwagwalada, Abuja, Nigeria; d Department of Medical Laboratory Science, University of Nigeria, Enugu Campus, Nigeria; e Department of Medical Laboratory Science, Faculty of Allied Health Sciences, Ahmadu Bello University, Zaria, Nigeria; f Department of Obstetrics and Gynaecology, Faculty of Medicine and Health Sciences, Universiti Putra Malaysia, 43400, Selangor, Malaysia; g Department of Medical Laboratory Science, Ebonyi State University, Abakaliki, Nigeria; h Department of Medical Laboratory Science, University of Jos, Jos, Nigeria; i Department of Nursing Science, Maryam Abacha American University of Niger, Maradi, Nigeria

**Keywords:** Protein-energy malnutrition, Maternal anemia, Hematological indices, Nigeria

## Abstract

**Background:**

Despite the efforts to encourage the intake of nutritional supplements during antenatal periods, there are still many cases of anemia and protein-energy malnutrition during pregnancy. Hence, this study determined the incidence of anemia, protein-energy malnutrition, and associated risk factors among pregnant women in Abuja, Nigeria.

**Materials and methods:**

This hospital-based, case-control study involved randomly selected 176 pregnant and non-pregnant women attending the University of Abuja Teaching Hospital (UATH), Gwagwalada, Nigeria. Hemoglobin and hematocrit measurements were used to determine anemia incidence, while plasma protein, zinc levels and body mass index (BMI) were used to determine energy index status. Complete blood counts were analyzed using 5 parts-automatic hemo-analyzer, while plasma protein and zinc were analyzed using calorimetric method. Anemia and protein-energy malnutrition were defined using the World Health Organization (WHO) cut-off values.

**Results:**

The mean age of participants was 28.75 ± 5.22 years. Out of 176 participants, 7 (4%) were malnourished while 25% of the participants were anemic. Anemia was significantly associated with participants’ occupation (p = 0.002), parity (p<0.001) and gestational age (p<0.001). Most hematological indices, plasma globulin, albumin, protein, and zinc levels were significantly different (p<0.001) among non-pregnant and pregnant women of the first, second and third trimesters.

**Conclusion:**

The incidence of anemia and malnutrition was high among study participants. There is a need for improved nutritional intervention, increased awareness and strengthening of health systems in the area of maternal health in Nigeria.

## 1. Introduction

Anemia in pregnancy is a global public health challenge in developing countries including Sub Saharan Africa, having the highest incidence of this condition with resultant effects on social and economic development [1]. Anemia during pregnancy is one of the common complications of pregnancy and a leading cause of maternal morbidity and mortality. The effect of maternal anemia is not restricted alone to the pregnant mother, but its negative consequence results to low birth weight, stillbirth, impaired cognitive development, obstructive labor among other poor perinatal outcomes [2]. Anemia in pregnancy poses a significant challenge to low middle income countries such as Nigeria with fragile healthcare systems. This maternal health emergency condition serves as an indicator that defines the socio-economic welfare of a country and has an enormous impact on the quality of life. Anemia in pregnancy is referred to as the common complication of pregnancy with potential effect to both mother and child, hence requires special attention from all stakeholders within and beyond the healthcare sector.

Several factors are known to be associated with anemia in pregnancy either as risk factors or predictors. These factors range from sociodemographic variables such as maternal age, parity, gravidity, gestational age at antenatal booking especially during the third trimester and occupation [2,3], anthropometric indices such as height, weight, waist-hip circumference and body mass index [4–9], nutritional and biochemical parameters (C-reactive protein, albumin, retinol, consumption of fruits, vegetables, red meat, eggs, beans, tea, zinc, vitamins, folate, iron, before and during pregnancy) [10,11]. Poor dietary patterns can predispose pregnant women and their fetus to lack essential nutrients leading to malnutrition, impaired cognitive capacity, increased susceptibility to infection and low productivity [12].

Apart from the adverse health of anemia on maternal and perinatal health, protein-energy malnutrition is also one of the major nutritional health problem that is common among women of reproductive age including pregnant mothers. When pregnant women lack energy and insufficient protein in their diet, they become deficient in micronutrients such as vitamins, ferritin, folate, and zinc which made both baby and mother to be susceptible to anemia and developmental defects [13]. To our knowledge, there is paucity of data on the incidence of anemia and protein-energy malnutrition in FCT-Abuja, Nigeria. Hence, this study was aimed to determine the incidence of anemia and protein-energy malnutrition and associated risk factors and to explore the relationship between malnutrition and the incidence of anemia among pregnant women in FCT-Abuja, Nigeria.

## 2. Materials and methods

### 2.1. Study area

This study was conducted at the University of Abuja Teaching Hospital, Gwagwalada, FCT-Abuja. Gwagwalada is one of the five local government areas of Abuja, the federal capital territory of Nigeria. Abuja is located at 9°4′0″N, 7°29′0″E in the North-central geopolitical region of Nigeria. It has an area of 1043 km^2^ with an estimated population of 3,464,123 as at 2021 [14]. The University of Abuja Teaching hospital records the average of 2401 deliveries on an annual basis and total of 9604 deliveries were recorded between January 1st, 2012 to December 31st, 2016 [15].

### 2.2. Study design

This study was a hospital-based case-control study involving pregnant women (case) attending the antenatal clinic of the University of Abuja Teaching Hospital and an apparently healthy non pregnant women (control). The control group were female students on clinical rounds and postings who voluntarily accepted to participate in the study.

### 2.3. Study population

A total of 176 participants were enrolled in the study, one hundred and eighteen (118) pregnant women and 58 non-pregnant women (controls) between the age of 15–40 years were recruited to participate in the study.

### 2.4. Participant's selection criteria

Inclusion criteria for the study case group were women of reproductive age who are between 15 and 40 years and receiving antenatal care services at the University of Abuja Teaching Hospital between the period April to December 2018. While age matched apparently healthy female medical students who were non pregnant were selected and served as control group for the study. Women with known clinical complications including bleeding disorders were excluded to participate in the study.

### 2.5. Anthropometric measurement

The heights of the subjects were measured using a calibrated Stadiometer, model 220 (manufactured by Seca Gmbh and Co., Germany). The subjects were weighed with minimum clothing to the nearest 0.1kg using a regularly calibrated health weighing scale; model ZT 120 (manufactured by Seca Gmbh and Co., Germany). Body mass index (BMI) was calculated using the expression: BMI (kg/m2) = Body weight (kg)/Height (m2) [18,19].

### 2.6. Ethical clearance and informed consent

Ethical approval was obtained from the University of Abuja Teaching Hospital Health Research Ethical Committee (UATH-HREC). Before the recruitment, written informed consent was obtained from all eligible participants before enlisting. The study was conducted in accordance with the standards of human experimentation as described in the Helsinki Declaration of 1975, as revised in 2000 [20].

### 2.7. Data collection

A validated semi-structured interviewer-administered questionnaire was used as an instrument of data collection. The questionnaire was used to obtain information on sociodemographic, anthropometric, clinical and nutritional information of the study participants.

### 2.8. Sample collection

Using a syringe and needle, about 5 mL of blood samples were asceptically collected into ethylenediamine-N,N,N,N-tetra acetate (EDTA) bottle and plain bottle respectively. Blood samples were transported to the laboratory under cold chain. The blood samples collected in plain container were allowed to clot and was centrifuged at 5000 rpm for 10 minutes and the clear non haemolysed serum were harvested and aliquot into cryovials and stored at 2–6 °C pending analysis.

## 3. Laboratory analytical protocol

### 3.1. Full hematology assay

Full blood counts and differential leucocytes counts were analyzed using Sysmex hematology automated analyzer (NY, USA) following the manufacturer's operational guidelines. All samples for hematological analysis were analyzed within 30 minutes of collection.

### 3.2. Anemia status determination

Anemia was termed as hematocrit level of <33% or hemoglobin concentration <11 g/dl. Mild anemia was termed as hematocrit level between 30 and 32.9% or hemoglobin concentration between 10 and 10.9 g/dl, moderate anemia as hematocrit level between 21 and 29.9% or hemoglobin 7 and 8.9 g/dl, while severe anemia was termed as a hematocrit level below 21% or hemoglobin concentration below 7 g/dl [16,17].

### 3.3. Nutritional and biochemical analysis

Serum Zinc levels was determined using atomic absorption spectrophotometric method while Serum total protein and albumin were determined using methods of Biuret [21] and Bromocresol green [22] respectively, while globulin was computed from the values generated from total protein and albumin. Per run and daily quality control for all laboratory procedures were ensured.

### 3.4. Statistical analysis

Data generated from the study were analyzed using statistical package for social sciences (SPSS) version 23 (SPSS Inc., Chicago, IL, USA). Data were presented as mean ± standard error of mean. Comparison of greater than two experimental groups was done using the one-way ANOVA and appropriate Least Significant Difference (LSD) post hoc test was done to determine mean difference among the groups. Chi-square test was used to test for association between categorical variables. Statistical significance was considered at p-value ≤ 0.05.

## 4. Results

The mean age of participants was 28.75 ± 5.22 years. Majority of the participants were civil servants as shown in Table 1. Out of 176 participants, 7 (4%) were malnourished while about a quarter (25%) of the participants were anemic. Out of the anemic pregnant women, 11.4% were at 1st trimester, 54.5% at 2nd trimester and 34.1% at 3rd trimester as demonstrated in Table 2. Out of the anemic pregnant women, 12/44 (27.3%) were moderately anemic, while 32/44 (72.7%) were mildly anemic. About 27.3% nulliparous women were anemic while 72.7% multiparous were anemic. There was significant (p<0.05) association between gestation age, parity and the incidence of anemia among pregnant women in the present study.

As shown in Table 3, Body mass index and types of food predominantly consumed by pregnant women were not associated with the incidence of anemia in the present study.

The mean ± SEM of the WBC (x10^9^ cells/mL) of non-pregnant women, pregnant women at 1st, 2nd and 3rd trimesters were 5.38 ± 0.17, 7.06 ± 0.31, 8.39 ± 0.27, and 7.51 ± 0.26, respectively. For the WBC absolute differentials, the mean ± SEM of the Lymphocyte (cells/mL) versus neutrophil counts (cells/mL) of non-pregnant women, pregnant women at 1st, 2nd and 3rd trimesters were 2.14 ± 0.08 versus 2.71 ± 0.15, 2.27 ± 0.13 versus 4.30 ± 0.29, 1.93 ± 0.06 versus 5.86 ± 0.25 and 1.83 ± 0.06 versus 5.05 ± 0.22, respectively.

The mean ± SEM of the RBC (x10^12^ cells/mL) of non-pregnant women and pregnant women at 1st, 2nd and 3rd trimesters were 4.98 ± 0.10, 4.33 ± 0.09, 3.83 ± 0.05, and 3.78 ± 0.07, respectively. Moreover, the mean ± SEM of the haemoglobin concentration (g/dl) of non-pregnant women, pregnant women at their 1st, 2nd and 3rd trimesters were 13.13 ± 0.14, 11.55 ± 0.18, 10.97 ± 0.14 and 10.77 ± 0.16, respectively. The mean ± SEM of the platelet count (x10^3^ cells/mL) of non-pregnant women and pregnant women at 1st, 2nd and 3rd trimesters were 214.9 ± 6.9, 228.6 ± 9.6, 237.4 ± 11.4, and 212.5 ± 9.2, respectively (Table 4).

There were significant mean differences in the hematological parameters (white blood cell count (WBC) (p = 0.00001), red blood cell count (RBC) (p = 0.00001), hemoglobin concentration (HB) (p = 0.00001), packed cell volume (PCV) (p = 0.00001), mean corpuscular volume (MCV) (p = 0.000), mean corpuscular hemoglobin (MCH) (p = 0.003), lymphocyte percentage (LYM %) (p = 0.000), monocyte percentage (MXD %) (p = 0.000), neutrophil percentage (NEUT %) (p = 0.000), lymphocyte count (LYM) (p = 0.001) & neutrophil count (NEUT) (p = 0.000)] among the four categories of subjects (i.e. non-pregnant, pregnant at 1st, 2nd and 3rd trimesters) (Table 4). Post-hoc analysis shows differences in mean ± SEM of these parameters varied between first trimester and non-pregnant women, 2nd trimester and non-pregnant women, 1st trimester and 2nd trimester, 1st trimester and 3rd trimester, 2nd trimester and 3rd trimester (Table 5).

The mean ± SEM of globulin level in non-pregnant women at 1st, 2nd, 3rd trimester were 30.15 ± 0.32g/L, 30.66 ± 0.66g/L, 32.93 ± 0.43g/L and 32.80 ± 0.52g/L respectively. There was a significant (p<0.0001) increase in the levels of plasma globulin from non-pregnant women, for pregnant women at 1st trimester and 3rd trimester as shown in Table 6. The mean ± SEM of albumin level in non-pregnant women at 1st, 2nd and 3rd trimester were 41.86 ± 0.29g/L, 38.79 ± 0.54g/L, 35.86 ± 0.23g/L and 35.15 ± 0.25g/L, respectively. There was a significant (p<0.0001) decrease in levels of serum albumin among non-pregnant women, pregnant women at 1st trimester to those at 3rd trimester.

The mean ± SEM of serum total protein levels among non-pregnant women, pregnant women at 1st, 2nd and 3rd trimester were 72.09 ± 0.44g/L, 69.45 ± 0.96g/L, 68.79 ± 0.48g/L and 67.96 ± 0.60g/L, respectively. There was a significant (p<0.0001) decrease in serum total protein levels among non-pregnant women, pregnant women at 1st, 2nd and 3rd trimester. The mean ± SEM of serum zinc concentration among non-pregnant women and pregnant women at 1st, 2nd and 3rd trimester were 20.49 ± 0.28 μmol/L, 20.61 ± 0.87 μmol/L, 21.79 ± 0.22 μmol/L and 21.94 ± 0.22 μmol/L, respectively. There was a significant (p<0.0001) increase in the concentration of serum zinc among non-pregnant women and pregnant women at 1st, 2nd and 3rd trimester. After post-hoc analysis, mean ± SEM difference of these parameters varied among first trimester and non-pregnant women, 2nd trimester and non-pregnant women, 1st trimester and 2nd trimester, 1st trimester and 3rd trimester, 2nd trimester and 3rd trimester as shown in Table 7.

## 5. Discussion

Anemia in pregnancy has continued to emerge as a public health concern due to its negative health consequences on maternal health; eclampsia, postpartum bleeding, postnatal bacterial infection, increased risk for preterm delivery, delivery of low-birth-weight babies, stillbirth among others [2].

The average age of participants (28.75 ± 5.22 years) in the present study was higher when compared with the findings from Sagamu, Ogun State, Nigeria where the mean age of participants was 25.4 ± 4.2 years [23]. The incidence of anemia in the present study was 25% (44/176). This was similar to findings reported by previous studies conducted in Enugu [28% (56/200)] [24] and India [20% (6/30)] [25]. The incidence of anemia as reported in the present study was higher than those reported in Zaria, Kaduna State, Nigeria [12.2% (11/90)] [26], and Gondar, North-West Ethiopia [16.6% (50/302)] [7]. The proportion of anemia in pregnant women in our study was much lower than findings from Sagamu [32.5% (130/400)] [23], Enugu [40.4% (214/530)] [24], Nepal [42.6% (368/863)] [27], Tanzania [47.4% (1257/2654)] [28], Southern Ethiopia [39.94% (145/363)] [29], Ibadan [30.2% (815/2702)] [30], Lagos [35.3% (132/374)] [31], South-South Nigeria [32.2% (388/1205)] [32], Oyo State [32.8% (196/597)] [4], Ilesha [62.2% 280/450)] [33], Abeokuta [76.5% (365/477)] [6], Gombe [51.8% (239/461)] [34], Pakistan [90.5% (1236/1366)] [10] and India [83% (62/75)] [35].

In the present study, we reported that about 3/4 [72.7% (32/44)] of anemic pregnant women were mildly anemic. This finding is in tandem with the previous studies in Nigeria; Sagamu, Ogun State [72.3% (94/130)] [23] and Gombe state [67.4% (161/239)] [36] as well as Pakistan [82.8% (1024/1236)] [10] and Gondar in Ethiopia [64% (32/50)] [27]. The proportion of those with mild anemia in our study was however lower than those compared to previous studies conducted in Enugu, Nigeria [90.7% (194/214)] [5], [94.6%, (53/56)] [24] and Nepal [90.8% (334/368)] [28]. On the other hand, the proportion of our study subjects with mild anemia was not just in the majority but was higher compared to those of previous studies conducted in Southern Ethiopia [29] and Abeokuta in Nigeria [6] which reported a comparatively lower incidence of mild anemia and proportions of moderate anemia.

On the other hand, low incidence of moderate anemia in pregnancy were reported in Northern Tanzania [9.9% (124/1257)] [8], Nepal [7.1% (26/368) [27], Pakistan [14.8% (183/1236)] [10], and Enugu [9.3% (20/214)] [15], [5.4%, (3/56)] [24] when compared to that reported in our study. Other studies conducted in Southern Ethiopia [60% (87/145)] and Abeokuta [57.8% (211/365)] [6] reported higher incidence of moderate anemia when compared to our study.

The absence of severe anemia cases as reported in our study is in line with study conducted in Enugu, Nigeria [15]. In contrast, the incidence of severe anemia in pregnancy were reported by previous studies [6,8,10,27,33,34]. The varying findings of the levels of anemia in pregnancy may be due to poor awareness and health education promotion practices during the prenatal clinics. Health education of pregnant mothers becomes necessary during the clinics where health workers could advice the mothers on the proper diets and nutrients including supplements to be taken during pregnancy with a view to prevent anemia in pregnancy can never be overemphasized.

In the present study, we found no association between sociodemographic variables and anemia. Maternal age had no association with anemia. This finding agrees with previous studies conducted in Enugu, Nigeria [24] and Shagamu, ogun State, Nigeria [23]. However, our finding differ with previous studies conducted in Oyo state, Nigeria [4,9] and in India [35] where significant associations were reported between maternal age and anemia. In contrast to the study conducted in Sagamu, Nigeria [23], our study observed a significant association between occupation and anemia. Our findings are however in line with the study conducted in Ethiopia [37] which reported a significant association between maternal occupation (farmer) and anemia in pregnancy. The status of occupation may be related to the lack of information on the adequate and appropriate attitude to achieve good nutritional practices during the state of pregnancy. Unlike pregnant women with non-manual jobs, those with manual jobs have been reported to be predisposed to longer periods of work, poor working conditions and low remuneration could predispose pregnant women to anemia due to lack of money to buy foods that could prevent anemia. These factors further subject those affected to unfavourable or inconsistent dietary habits [38]. Lack of rest and poor sleeping patterns have also been reported to be associated with anemia [39].

Based on the association between maternal clinical characteristics and anemia, our study found a strong association between parity and anemia which agrees with some previous studies [9,24,29,33] but in contrast with other studies [5,34], our study reported that gestational age was significantly associated with anemia. This is in line with previous studies conducted in Enugu [5], Tanzania [28], Southern Malawi [40] and Ghana [41] where it was reported that anemia at booking varies direct proportionally with gestational age. This implies that pregnant women who fail to book on time for antenatal care are usually at a higher risk of developing anemia compared to those that booked earlier within their first or second trimester. Physiologic hemodilution in pregnancy has been associated with an elevated proportion of mild anemia. The levels of hemoglobin and packed cell volume (PCV) have been reported to decrease steadily as plasma volume increases during the first trimester till extreme levels are attained at the end of the second trimester. During the third trimester, hemoglobin and PCV begins to increase as plasma volume decreases [42]. This further implies that pregnant women in their second trimester are prone to being anemic than their counterparts at third trimester. This was reported in our study, pregnant women in their second trimester (54.5%) had a higher incidence of anemia compared to their counterparts in at first (11.4%) and third (34.1%) trimesters. The decrease in the concentration of hemoglobin could have negative consequence on both maternal and fetal health if urgent intervention is not instituted. Our findings are in conformity with previous studies conducted in Ghana [41] and India [25]. In contrast to our findings, a study conducted in Zimbabwe reported that hemoglobin concentration was independent of gestational age and season of sampling, hence these factors are not significant predictors of anemia in pregnancy [43]. Our study also reported a strong positive association between parity and anemia. On the other hand, previous findings reported contrary results [30,36,44]. This suggests that as parity increases, the likelihood of pregnant mother to become anemic while pregnant increased. This could be due to the consistent drain of iron stores in the body [45].

In contrast to our study on the association between body mass index (BMI) as well as diet restriction/adherence and anemia as shown in Table 3, previous studies conducted in Mexico [46] and Indonesia [47] reported an association between anemia based on hemoglobin levels and BMI due to nutritional status and appropriate consumption of meals and nutritional supplements. The underweight proportion of participants (malnourished) in our study was 4% (7/176) which conforms with the previous study conducted in Ebonyi State, Nigeria [5.9% (18/305)] [48]. The proportion (4.5%) of participants who were both malnourished and anemic were higher compared to those who were non-anemic but malnourished (3.8%). This could suggest that malnutrition, which although was not demonstrated to be associated with anemia in our study, may have the potential to increase the risk of pregnant women to anemia compared to those with normal weight (not malnourished) as observed in our study having reported lower proportion (15.9%) of normal weight subjects who are anemic compared to those that are non-anemic (22.7%). Pregnant women who are malnourished lack energy, essential amino acids, vitamins and a range of vital micronutrients (e.g., folate and iron) which predisposes them to anemia and can lead to the delivery of anemic infants with decreased mental capacity, psychomotor disorders and behavioral defects [46,49,50].

All hematological parameters in our study except for MCHC, MXD, RDW-SD and RDW-CV significantly varied among the stages of pregnancy. These findings were not similar to those reported in Sagamu, South-West, Nigeria [51], which found no significant differences in most of the hematological parameters (such as WBC, eosinophil, basophils, neutrophils, RBC) between pregnant and non-pregnant women. This could be the result of the differences in sample size and laboratory test protocols used in our study.

The WBCs were observed in our study to be significantly increased with increase in gestational age of study participants as observed in a previous study [52]. The sustained increase in total WBC may be due to the buildup of fetal immunity which is attained by a process of selective tolerance and immunomodulation in the presence of increased immunity against microbes. To enhance maternal adaptation during the stages of pregnancy, immune responses from cytotoxic T-cells are suppressed, while certain immune cells including the neutrophils are activated [53].

Following what was observed in a previous study [52], neutrophils accounted for the elevated total WBC as observed in our study but the levels of this innate immune cell were elevated throughout the stages of pregnancy. Similarly, we observed in our study that monocytes were activated and observed to be elevated throughout the stages of pregnancy, while lymphocyte counts were observed to be significantly reduced with an increase in gestational age. These observations agreed with previous studies [52,53].

In our study, PCV, RBC and HB were observed to be significantly decreased with an increase in gestational age, which although agreed with the study [23] but reported that PCV was not influenced by the stages of pregnancy [51]. The decrease in haematological indices during pregnancy as reported in the present study may be due to elevated plasma volume during pregnancy which leads to hemo-dilution with resultant hormonal alterations that induce fluid retention and deficiency of iron [54–56]. Besides these contributing factors, poor health system indicated by low quality antenatal healthcare services, inconsistency in patterns of consuming diet and nutritional supplements may lead to the pattern of these hematological parameters as observed in our study.

After comparison of the platelet counts between pregnant and non-pregnant women, we found an increase in mean platelet count (PLT) in the pregnant women when compared with the non-pregnant women. However, these changes were not statistically significant (p>0.05). In pregnancy, increased platelet destruction may be mediated by immunological mechanisms, abnormal platelet activation, or platelet consumption [52].

Besides the hematological indices, our study also aimed at investigating the influence of the stages of pregnancy on both nutritional and biochemical indices. All parameters were observed to statistically vary with an increase in gestational age. With exception of the serum levels of total protein and albumin, levels of globulin and zinc were observed to increase as the pregnancy progressed. Similar pattern was reported by previous work [57]. The observed decreased in serum levels of albumin (hypoalbuminemia) in our study may not only be due to decreased production but also due to hemodilution (i.e., resulting from hypervolemia) and proteinuria.

During the onset of pregnancy, plasma globulin is observed at to be low due to inhibition of T-cell-mediated immune response and disintegration of tryptophan by maternal dendritic cells and the suppression of pro-inflammatory cytokines including interleukin-12, tumor necrosis factor-alpha and interferon-gamma. These adjustments are necessary to accommodate pregnancy at the expense of increased susceptibility to infection [58,59]. These changes could be responsible for the initial insignificant decrease in serum globulin levels during the second trimesters of respondents in comparison to that of their counterparts in their third trimesters as observed in our study.

Consequently, the present study found an increased levels of serum zinc which increase as the pregnancy progresses. Zinc is regarded as one of the strongest predictors of haemoglobin-based on their positive relationship [11,60–62] as it is involved in hemoglobin synthesis through the function of certain zinc-dependent enzyme systems which include aminolevulinic acid dehydrase required for heme synthesis [63], thymidine kinase required for the synthesis of DNA and GATA-1 (zinc transcription factor) for physiological erythropoiesis [64]. Other roles of zinc have been observed in cell membrane stabilization [65] and in enhancing insulin-like growth factor-1 levels which facilitate hematopoiesis [66]. Despite these haemoglobin-enhancing roles of zinc, it was expected that serum zinc levels ought to have decreased as the pregnancy advances. However, the possible reason for the increased zinc with the gestational age of our study participants could be due to the increased awareness during antenatal visits in the value of nutritional supplements which include zinc as well as good dietary use by our study subjects having indicated that greater number of pregnant women were not malnourished in the present study.

Moreover, our study may be prone to selection bias due to the location of the study area, as the participants reside in urban settlement, Abuja, Nigeria. Due to fund constraint the study could not include other pregnant women from the rural areas.

## 6. Conclusion

The incidence of anemia and malnutrition among pregnant women in Gwagwalada was on the increase. Occupation, gestational age, and parity were associated with anemia in pregnancy. The stages of pregnancy were observed to influence the nutritional, biochemical and hematological parameters in the present study. Hence, the need for improved nutritional intervention, health education, increased awareness and strengthening health systems in the area of maternal health in Nigeria.

## Figures and Tables

**Table 1:** Sociodemographic characteristics of study participants and their anemic status.

Variables	Characteristics	Pregnant n (%)	Non-pregnant n (%)
Age (years)	15–20	0 (0)	10 (17.2)
	21–25	13 (11.0)	23 (39.7)
	26–30	47 (39.8)	14 (24.1)
	31–35	38 (32.2)	8 (13.8)
	36–40	20 (16.9)	3 (5.2)
Occupation	Student	5 (4.2)	3 (5.2)
	Cleaner	2 (1.7)	0 (0.0)
	Civil servant	15 (7.9)	55 (94.8)
	Trader	47 (39.8)	0 (0.0)
	Volunteer worker	2 (1.7)	0 (0.0)
	Housewife	21 (17.8)	0 (0.0)
	Teacher	11 (9.3)	0 (0.0)
	Stylist	2 (1.7)	0 (0.0)
	Accountant	1 (0.8)	0 (0.0)
	Banker	3 (2.5)	0 (0.0)
	Tailor	3 (2.5)	0 (0.0)
	Caterer	1 (0.8)	0 (0.0)
	Applicant	1 (0.8)	0 (0.0)
	Cook	1 (0.8)	0 (0.0)
	Self-employed	1 (0.8)	0 (0.0)
	Site manager	1 (0.8)	0 (0.0)
	Public servant	1 (0.8)	0 (0.0)
Anemia Status	Yes	44 (37.3)	0 (0.0)
	No	74 (62.7)	58 (100.0)

**Table 2:** Association of some clinical features and anemic status among study participants.

Variable	Indices	Anemic (n = 44)	Non-anemic (n = 132)	χ^2^	p-value
Gestational age	Zero trimester	0 (0)	58 (43.9)	33.744	<0.001*
	First trimester	5 (11.4)	17 (12.9)		
	Second trimester	24 (54.5)	28 (21.2)		
	Third trimester	15 (34.1)	29 (22)		
Parity	Nulliparous (no birth)	0 (0)	34 (25.8)	15.161	0.001*
	Primiparous (1 birth)	12 (27.3)	35 (26.5)		
	Multiparous (2 or more births)	32 (72.7)	63 (47.7)		

Where

* = Significant level of p-value were considered at p ≤ 0.05.

**Table 3:** Association of nutritional indices and anemic status of the study participants.

Indices	Anemic (n = 44)	Non-anemic (n = 132)	Test statistic	

			χ^2^	*p*-value
BMI (Kg/m^2^)				
Underweight (18.5–20.9)	2 (4.5)	5 (3.8)	2.649	0.754
Normal weight (21–24.9)	7 (15.9)	30 (22.7)		
Overweight (25–29.9)	19 (43.2)	50 (37.9)		
Class 1 obesity (30–34.9)	9 (20.5)	34 (25.8)		
Class 2 obesity (35–39.9)	6 (13.6)	12 (9.1)		
Class 3 obesity (≥40)	1 (2.3)	1 (0.8)		
Meat				
No	6 (13.6)	9 (6.8)	1.793	0.181
Yes	38 (86.4)	123 (93.2)		
Vegetables/fruits				
No	3 (6.8)	6 (4.5)	0.331	0.565
Yes	41 (93.2)	126 (95.5)		
Beans				
No	4 (9.1)	10 (7.6)	0.101	0.751
Yes	40 (90.9)	122 (92.4)		
Rice				
No	3 (6.8)	2 (1.5)	2.835	0.092
Yes	41 (93.2)	130 (98.5)		
Milk				
No	5 (11.4)	12 (9.1)	0.189	0.664
Yes	39 (88.6)	120 (90.9)		
Fish				
No	2 (4.5)	7 (5.3)	0.040	0.841
Yes	42 (95.5)	125 (94.7)		
Egg				
No	6 (13.6)	12 (9.1)	0.702	0.402
Yes	38 (86.4)	120 (90.9)		

Where

* = Significant level of p-value were considered at p ≤ 0.05.

**Table 4:** Comparison of maternal hematological profile at various trimesters with non-pregnant control mothers.

Hematological parameters	Non-pregnant (n = 58)	First trimester (n = 22)	Second trimester (n = 52)	Third trimester (n = 44)	*p*-value
WBC (×10^9^ cells/μL)	5.38 ± 0.17	7.06 ± 0.31	8.39 ± 0.27	7.51 ± 0.26	<0.0001*
RBC (×10^12^ cells/μL)	4.98 ± 0.10	4.33 ± 0.09	3.83 ± 0.05	3.78 ± 0.07	<0.0001*
HB (g/dl)	13.13 ± 0.14	11.55 ± 0.18	10.97 ± 0.14	10.77 ± 0.16	<0.0001*
PCV (%)	38.93 ± 0.37	34.42 ± 0.42	32.41 ± 0.35	32.08 ± 0.45	<0.0001*
MCV (fL)	79.86 ± 1.14	80.00 ± 1.33	85.03 ± 0.97	85.29 ± 1.01	<0.0001*
MCH (pg)	27.23 ± 0.33	26.85 ± 0.60	28.80 ± 0.44	28.66 ± 0.41	0.003*
MCHC (g/dL)	33.39 ± 0.23	33.53 ± 0.31	33.82 ± 0.18	33.56 ± 0.16	0.477
LYM (%)	40.08 ± 1.41	32.93 ± 2.01	24.56 ± 0.75	25.04 ± 0.92	<0.0001*
MXD (%)	10.07 ± 0.57	6.72 ± 0.43	6.59 ± 0.27	8.50 ± 0.41	<0.0001*
NEUT (%)	49.13 ± 1.37	60.33 ± 2.17	68.90 ± 0.85	64.51 ± 2.53	<0.0001*
LYM (cells/μL)	2.14 ± 0.08	2.27 ± 0.13	1.93 ± 0.06	1.83 ± 0.06	0.001*
MXD (cells/μL)	0.54 ± 0.04	0.49 ± 0.04	0.56 ± 0.03	0.63 ± 0.03	0.091
NEUT (cells/μL)	2.71 ± 0.15	4.30 ± 0.29	5.86 ± 0.25	5.05 ± 0.22	<0.0001*
RDW-SD (fL)	42.18 ± 0.69	42.03 ± 0.73	40.39 ± 2.17	43.71 ± 0.55	0.382
RDW-CV (%)	14.41 ± 0.38	14.98 ± 0.32	14.24 ± 0.17	14.50 ± 0.20	0.537
PLT (×10^3^ cells/μL)	214.9 ± 6.9	228.6 ± 9.6	237.4 ± 11.4	212.5 ± 9.2	0.1837

Data were presented as mean ± SEM;

* = Significant level of p-value were considered at p ≤ 0.05.

**Table 5:** Comparison of mean values of maternal hematological parameters at various of pregnancy and non-pregnant control women using the least significant difference (LSD) post hoc.

Parameter	Groups		Mean Difference	P-value

	Non-pregnant state	First trimester		
WBC (×10^9^ cells/μL)	5.38 ± 0.17	7.06 ± 0.31	−1.68 ± 0.41	<0.0001*
RBC (×10^12^ cells/μL)	4.98 ± 0.10	4.33 ± 0.09	0.65 ± 0.14	<0.0001*
HB (g/dl)	13.13 ± 0.14	11.55 ± 0.18	1.58 ± 0.26	<0.0001*
PCV (%)	38.93 ± 0.37	34.42 ± 0.42	4.51 ± 0.68	<0.0001*
MCV (fL)	79.86 ± 1.14	80.00 ± 1.33	−0.13 ± 1.86	0.943
MCH (pg)	27.23 ± 0.33	26.85 ± 0.60	0.37 ± 0.71	0.595
LYM (%)	40.08 ± 1.41	32.93 ± 2.01	7.15 ± 2.05	0.001*
MXD (%)	10.07 ± 0.57	6.72 ± 0.43	3.35 ± 0.78	<0.0001*
NEUT (%)	49.13 ± 1.37	60.33 ± 2.17	−11.19 ± 2.86	<0.0001*
LYM	2.14 ± 0.08	2.27 ± 0.13	−0.13 ± 0.13	0.317
NEUT	2.71 ± 0.15	4.30 ± 0.29	−1.59 ± 0.36	<0.0001*
PLT (×10^3^ cells/μL)	214.9 ± 6.9	228.6 ± 9.6	−13.7 ± 2.7	0.806
	Non-pregnant state	Second trimester		
WBC (×10^9^ cells/μL)	5.38 ± 0.17	8.39 ± 0.27	−3.01 ± 0.32	<0.0001*
RBC (×10^12^ cells/μL)	4.98 ± 0.10	3.83 ± 0.05	1.15 ± 0.10	<0.0001*
HB (g/dl)	13.13 ± 0.14	10.97 ± 0.14	2.16 ± 0.20	<0.0001*
PCV (%)	38.93 ± 0.37	32.41 ± 0.35	6.52 ± 0.52	<0.0001*
MCV (fL)	79.86 ± 1.14	85.03 ± 0.97	−5.17 ± 1.42	<0.0001*
MCH (pg)	27.23 ± 0.33	28.80 ± 0.44	−1.57 ± 0.54	0.004*
LYM (%)	40.08 ± 1.41	24.56 ± 0.75	15.52 ± 1.56	<0.0001*
MXD (%)	10.07 ± 0.57	6.59 ± 0.27	3.48 ± 0.59	<0.0001*
NEUT (%)	49.13 ± 1.37	68.90 ± 0.85	−19.77 ± 2.18	<0.0001*
LYM	2.14 ± 0.08	1.93 ± 0.06	0.21 ± 0.10	0.030*
NEUT	2.71 ± 0.15	5.86 ± 0.25	−3.15 ± 0.28	<0.0001*
PLT (×10^3^ cells/μL)	214.9 ± 6.9	237.7 ± 11.4	−22.8 ± 4.5	0.259
	Non-pregnant state	Third trimester		
WBC (×10^9^ cells/μL)	5.38 ± 0.17	7.51 ± 0.26	−2.13 ± 0.33	<0.0001*
RBC (×10^12^ cells/μL)	4.98 ± 0.10	3.78 ± 0.07	1.20 ± 0.11	<0.0001*
HB (g/dl)	13.13 ± 0.14	10.77 ± 0.16	2.35 ± 0.21	<0.0001*
PCV (%)	38.93 ± 0.37	32.08 ± 0.45	6.84 ± 0.54	<0.0001*
MCV (fL)	79.86 ± 1.14	85.29 ± 1.01	−5.43 ± 1.49	<0.0001*
MCH (pg)	27.23 ± 0.33	28.66 ± 0.41	−1.43 ± 0.57	0.012*
LYM (%)	40.08 ± 1.41	25.04 ± 0.92	15.04 ± 1.64	<0.0001*
MXD (%)	10.07 ± 0.57	8.50 ± 0.41	1.58 ± 0.62	0.012*
NEUT (%)	49.13 ± 1.37	64.51 ± 2.53	−15.37 ± 2.28	<0.0001*
LYM	2.14 ± 0.08	1.83 ± 0.06	0.32 ± 0.10	0.002*
NEUT	2.71 ± 0.15	5.05 ± 0.22	−2.34 ± 0.29	<0.0001*
PLT (×10^3^ cells/μL)	214.9 ± 6.9	212.3 ± 9.2	2.6 ± 2.6	0.899
	First trimester	Second trimester		
WBC (×10^9^ cells/μL)	7.06 ± 0.31	8.39 ± 0.27	−1.33 ± 0.42	0.002*
RBC (×10^12^ cells/μL)	4.33 ± 0.09	3.83 ± 0.05	0.50 ± 0.14	<0.0001*
HB (g/dl)	11.55 ± 0.18	10.97 ± 0.14	0.57 ± 0.26	0.031*
PCV (%)	34.42 ± 0.42	32.41 ± 0.35	2.01 ± 0.69	0.004*
MCV (fL)	80.00 ± 1.33	85.03 ± 0.97	−5.03 ± 1.90	0.008*
MCH (pg)	26.85 ± 0.60	28.80 ± 0.44	−1.95 ± 0.72	0.007*
LYM (%)	32.93 ± 2.01	24.56 ± 0.75	8.37 ± 2.08	<0.0001*
MXD (%)	6.72 ± 0.43	6.59 ± 0.27	0.13 ± 0.79	0.869
NEUT (%)	60.33 ± 2.17	68.90 ± 0.85	−8.57 ± 2.90	0.004*
LYM	2.27 ± 0.13	1.93 ± 0.06	0.34 ± 0.13	0.009*
NEUT	4.30 ± 0.29	5.86 ± 0.25	−1.56 ± 0.37	<0.0001*
PLT (×10^3^ cells/μL)	228.6 ± 9.6	237.4 ± 11.4	−8.8 ± 1.5	0.899
	First trimester	Third trimester		
WBC (×10^9^ cells/μL)	7.06 ± 0.31	7.51 ± 0.26	−0.45 ± 0.43	0.298
RBC (×10^12^ cells/μL)	4.33 ± 0.09	3.78 ± 0.07	0.55 ± 0.14	<0.0001*
HB (g/dl)	11.55 ± 0.18	10.77 ± 0.16	0.76 ± 0.27	0.005*
PCV (%)	34.42 ± 0.42	32.08 ± 0.45	2.33 ± 0.71	0.001*
MCV (fL)	80.00 ± 1.33	85.29 ± 1.01	−5.30 ± 1.94	0.007*
MCH (pg)	26.85 ± 0.60	28.66 ± 0.41	−1.81 ± 0.74	0.015*
LYM (%)	32.93 ± 2.01	25.04 ± 0.92	7.90 ± 2.14	<0.0001*
MXD (%)	6.72 ± 0.43	8.50 ± 0.41	−1.77 ± 0.81	0.030*
NEUT (%)	60.33 ± 2.17	64.51 ± 2.53	−4.18 ± 2.98	0.163*
LYM	2.27 ± 0.13	1.83 ± 0.06	0.45 ± 0.13	0.001*
NEUT	4.30 ± 0.29	5.05 ± 0.22	−0.75 ± 0.38	0.049*
PLT (×10^3^ cells/μL)	228.6 ± 11.4	212.3 ± 9.2	16.3 ± 2.2	0.743
	Second trimester	Third trimester		
WBC (×10^9^ cells/μL)	8.39 ± 0.27	7.51 ± 0.26	0.88 ± 0.34	0.010*
RBC (×10^12^ cells/μL)	3.83 ± 0.05	3.78 ± 0.07	0.05 ± 0.11	0.675
HB (g/dl)	10.97 ± 0.14	10.77 ± 0.16	0.19 ± 0.21	0.363
PCV (%)	32.41 ± 0.35	32.08 ± 0.45	0.32 ± 0.56	0.555
MCV (fL)	85.03 ± 0.97	85.29 ± 1.01	−0.25 ± 1.52	0.867
MCH (pg)	28.80 ± 0.44	28.66 ± 0.41	0.14 ± 0.58	0.806
LYM (%)	24.56 ± 0.75	25.04 ± 0.92	−0.48 ± 1.68	0.776
MXD (%)	6.59 ± 0.27	8.50 ± 0.41	−1.90 ± 0.64	0.003*
NEUT (%)	68.90 ± 0.85	64.51 ± 2.53	4.39 ± 2.34	0.062
LYM	1.93 ± 0.06	1.83 ± 0.06	0.11 ± 0.10	0.313
NEUT	5.86 ± 0.25	5.05 ± 0.22	0.81 ± 0.30	0.007*
PLT (×10^3^ cells/μL)	237.4 ± 11.4	212.3 ± 9.3	25.1 ± 2.1	0.232

Data were presented as mean ± SEM;

* = Significant level of p-value were considered at p ≤ 0.05.

**Table 6:** Comparison of maternal biochemical profile at various trimesters and non pregnant control women.

Parameters	Non-pregnant (n = 58)	Pregnant			p-value

		First trimester (n = 22)	Second trimester (n = 52)	Third trimester (n = 44)	
Globulin (g/L)	30.15 ± 0.32	30.66 ± 0.66	32.93 ± 0.43	32.80 ± 0.52	<0.0001*
Albumin (g/L)	41.86 ± 0.29	38.79 ± 0.54	35.86 ± 0.23	35.15 ± 0.25	<0.0001*
Protein (g/L)	72.09 ± 0.44	69.45 ± 0.96	68.79 ± 0.48	67.96 ± 0.60	<0.0001*
Zinc (μmol/L)	20.49 ± 0.28	20.61 ± 0.87	21.79 ± 0.22	21.94 ± 0.22	0.001*

Data were presented as mean ± SEM;

* = Significant level of p-value were considered at p ≤ 0.05.

**Table 7:** Comparison of mean globulin, albumin, total protein and zinc levels of pregnant and non-pregnant mothers using the least significant difference (LSD) post hoc.

Parameter	Groups		Mean Difference	p-value

	Non-pregnant mothers	First trimester		
Globulin (g/L)	30.15 ± 0.32	30.66 ± 0.66	−0.51 ± 0.75	0.499
Albumin (g/L)	41.86 ± 0.29	38.79 ± 0.54	3.07 ± 0.50	<0.0001*
Protein (g/L)	72.09 ± 0.44	69.45 ± 0.96	2.64 ± 0.93	0.005*
Zinc (μmol/L)	20.49 ± 0.28	20.61 ± 0.87	−0.12 ± 0.55	0.827
	Non-pregnant mothers	Second trimester		
Globulin (g/L)	30.15 ± 0.32	32.93 ± 0.43	−2.78 ± 0.57	<0.0001*
Albumin (g/L)	41.86 ± 0.29	35.86 ± 0.23	6.00 ± 0.38	<0.0001*
Protein (g/L)	72.09 ± 0.44	68.79 ± 0.48	3.30 ± 0.71	<0.0001*
Zinc (μmol/L)	20.49 ± 0.28	21.79 ± 0.22	−1.29 ± 0.42	0.002*
	Non-pregnant mothers	Third trimester		
Globulin (g/L)	30.15 ± 0.32	32.80 ± 0.52	−2.65 ± 0.60	<0.0001*
Albumin (g/L)	41.86 ± 0.29	35.15 ± 0.25	6.70 ± 0.40	<0.0001*
Protein (g/L)	72.09 ± 0.44	67.96 ± 0.60	4.13 ± 0.74	<0.0001*
Zinc (μmol/L)	20.49 ± 0.28	21.94 ± 0.22	−1.45 ± 0.44	0.001*
	First trimester	Second trimester		
Globulin (g/L)	30.66 ± 0.66	32.93 ± 0.43	−2.27 ± 0.76	0.003*
Albumin (g/L)	38.79 ± 0.54	35.86 ± 0.23	2.93 ± 0.51	<0.0001*
Protein (g/L)	69.45 ± 0.96	68.79 ± 0.48	0.65 ± 0.95	0.487
Zinc (μmol/L)	20.61 ± 0.87	21.79 ± 0.22	−1.18 ± 0.56	0.036*
	First trimester	Third trimester		
Globulin (g/L)	30.66 ± 0.66	32.80 ± 0.52	−2.15 ± 0.78	0.007*
Albumin (g/L)	38.79 ± 0.54	35.15 ± 0.25	3.64 ± 0.52	<0.0001*
Protein (g/L)	69.45 ± 0.96	67.96 ± 0.60	1.49 ± 0.97	0.127
Zinc (μmol/L)	20.61 ± 0.87	21.94 ± 0.22	−1.33 ± 0.57	0.021*
	Second trimester	Third trimester		
Globulin (g/L)	32.93 ± 0.43	32.80 ± 0.52	0.12 ± 0.61	0.839
Albumin (g/L)	35.86 ± 0.23	35.15 ± 0.25	0.71 ± 0.41	0.085
Protein (g/L)	68.79 ± 0.48	67.96 ± 0.60	0.83 ± 0.76	0.277
Zinc (μmol/L)	21.79 ± 0.22	21.94 ± 0.22	−0.15 ± 0.45	0.734

Data were presented as mean ± SEM;

* = Significant level of p-value were considered at p ≤ 0.05.
